# The Effects of Vitamin D Supplementation on Anthropometric and Biochemical Indices in Patients With Non-alcoholic Fatty Liver Disease: A Systematic Review and Meta-analysis

**DOI:** 10.3389/fphar.2021.732496

**Published:** 2021-11-03

**Authors:** Shahla Rezaei, Reza Tabrizi, Peyman Nowrouzi-Sohrabi, Mohammad Jalali, Mojtaba Shabani-Borujeni, Shayan Modaresi, Maryam Gholamalizadeh, Saeid Doaei

**Affiliations:** ^1^ Nutrition Research Center, School of Nutrition and Food Sciences, Shiraz University of Medical Sciences, Shiraz, Iran; ^2^ Student Research Committee, Shiraz University of Medical Sciences, Shiraz, Iran; ^3^ Non-Communicable Diseases Research Center, Fasa University of Medical Sciences, Fasa, Iran; ^4^ Department of Biochemistry, School of Medicine, Shiraz University of Medical Sciences, Shiraz, Iran; ^5^ Nutrition Research Center, Student Research Committee, Shiraz University of Medical Sciences, Shiraz, Iran; ^6^ Department of Clinical Pharmacy, Faculty of Pharmacy, Shiraz University of Medical Sciences, Shiraz, Iran; ^7^ Student Research Committee, Cancer Research Center, Shahid Beheshti University of Medical Sciences, Tehran, Iran; ^8^ Research Center of Health and Enviroment, School of Health, Guilan University of Medical Sciences, Rasht, Iran

**Keywords:** vitamin D, NAFLD, liver enzyme, anthropometry, biochemical indices

## Abstract

**Background:** Vitamin D was reported to be associated with non−alcoholic fatty liver disease (NAFLD). This systematic review and meta−analysis aimed to investigate the effects of the vitamin D supplementation on anthropometric and biochemical indices in patient with NAFLD.

**Methods:** PubMed, Web of science, Scopus, and Embase databases were explored to identify all randomized controlled trial (RCT) investigating the effects of vitamin D supplementation on anthropometric and biochemical indices in patients with NAFLD. A random−effects model was used to pool weighted mean difference (WMD) and corresponding 95% confidence intervals (CIs). The statistical heterogeneity among the studies was assessed using I2 statistic (high ≥ 50%, low < 50%) and Cochran’s Q−test.

**Results:** Sixteen RCTs were included in this meta−analysis. The results identified that high−density lipoprotein−cholesterol (HDL−C) level significantly increased following vitamin D supplementation (*P* = 0.008). Vitamin D reduced body weight (*P* = 0.007), body mass index (*P* = 0.002), waist circumstance (WC) (*P* = 0.02), serum alanine transaminase (ALT) (*P* = 0.01), fasting blood sugar (FBS) (*P* = 0.01), homeostatic model assessment for insulin resistance (HOMA−IR) (*P* = 0.004), and calcium (*P* = 0.01). No significant changes were found on body fat, triglyceride (TG), total cholesterol, low−density lipoprotein−cholesterol (LDL−C), aspartate transaminase, alkaline phosphatase, gamma−glutamyl transferase, and adiponectin following vitamin D supplementation.

**Conclusion:** Vitamin D had significant effects on anthropometric and biochemical indices including HDL−C, body weight, BMI, WC, serum ALT, serum FBS, HOMA−IR, and calcium. Vitamin D supplementation can be considered as an effective strategy in management of patients with NAFLD.

**Systematic Review Registration**: [website], identifier [registration number]

## Background

Non-alcoholic fatty liver disease (NAFLD) is considered to be the most common cause of chronic liver disorders (Younossi, 2019). NAFLD includes a wide range of liver diseases such as fibrosis, cirrhosis and non−alcoholic hepatitis (Petta et al., 2016; Morvaridzadeh et al., 2021). The prevalence of NAFLD is about 20% in general population and about 70–90% in patients with type 2 diabetes (Tolman et al., 2007; Chalasani et al., 2012). The main causes of NAFLD are obesity, excessive dietary fat intake, insulin resistance, and dyslipidemia (Ludwig et al., 1980). Over the past few decades, various pharmacological and nutritional interventions have been assessed to treat NAFLD, but none indicated significant improvements. Currently, no medically approved drugs are available for NAFLD (Del Ben et al., 2014). Weight loss and nutritional interventions are considered standard treatments for NAFLD. Insulin resistance increases the rate of adipose tissue lipolysis and the flow of free fatty acids to the liver cell. Hyperglycemia also causes lipid−related changes in the liver cells by increasing lipogenesis while blocking fatty acid oxidation (FAO) and lipid transport in the liver (Kleiner et al., 2005; Schwimmer et al., 2005).

Vitamin D plays an important role in reducing insulin resistance, obesity, cardiovascular risk, prediabetes, metabolic syndrome, cancer, and cardiovascular diseases (CVD_s_) (Sepidarkish et al., 2019; Gholamalizadeh et al., 2020). Low serum level of 25-hydroxyvitamin D [25 (OH) D] is reported to be a risk factor for NAFLD disease (Forouhi et al., 2008; Sepidarkish et al., 2019). Vitamin D hypovitaminosis is associated with the severity and incidence of NAFLD in patients with normal liver enzymes. Vitamin D can affect the liver function through the vitamin D receptor (VDR). VDR is naturally present in the liver cells and its higher expression can reduce inflammation in chronic liver diseases (Benetti et al., 2018). Vitamin D also has anti−fibrotic, proliferative, and inflammatory effects on the liver. Some studies on the effects of vitamin D on anthropometric and biochemical indices reported that vitamin D was associated with body weight, fasting blood sugar (FBS), homeostatic model assessment for insulin resistance (HOMA−IR), glucose homeostasis, insulin resistance, high−density lipoprotein−cholesterol (HDL-C), low-density lipoprotein-cholesterol (LDL-C), triglycerides (TG), total cholesterol (TC), liver enzyme, and adiponectin (Sarathy et al., 2015; Foroughi et al., 2016; Doaei et al., 2019). A recent systematic review also reported that vitamin D improves the level of inflammatory mediators in patients with nonalcoholic fatty liver disease, but has no effect on anthropometric and glycemic indexes (Hariri and Zohdi, 2019; Mehrdad et al., 2020). However, the findings on the effects of vitamin D on NAFLD were contradictory (Sharifi et al., 2014; Sarathy et al., 2015). Therefore, this study aimed to investigate the effect of Vitamin D supplementation on anthropometric and biochemical indices in patient with NAFLD.

## Methods

**FIGURE 1:**
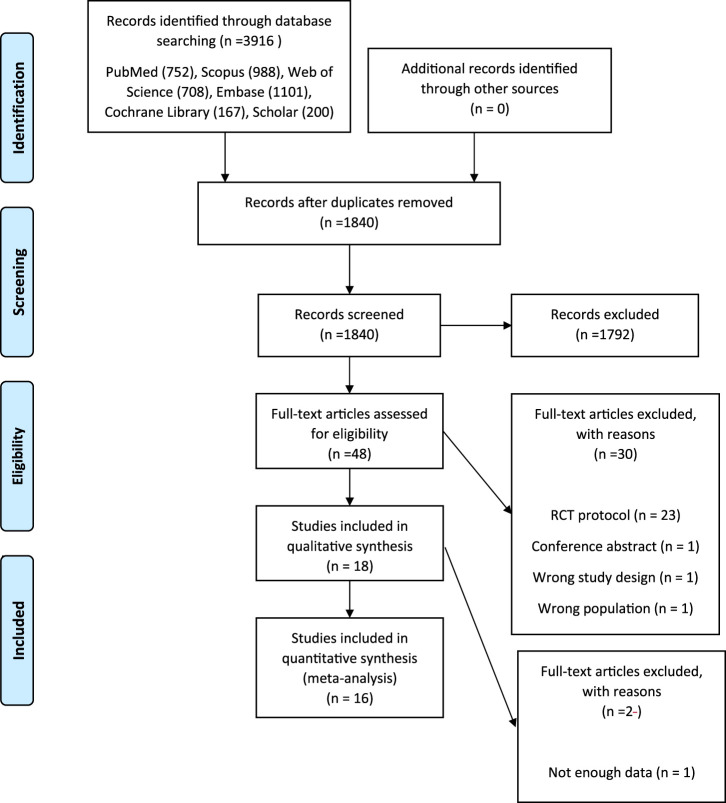
Flow chart of the literature search strategy and study selection.

Preferred reporting items for systematic reviews and meta−analyses (PRISMA) (Moher et al., 2009) was used to demonstrate the process of study selection (Figure 1).

### Search Strategy

Two independent researchers performed the process of the systematic search using the online databases such as PubMed, embase, Scopus, Web of Science, and Cochrane Library for publications from 1980 up to 2021 on the effects of vitamin D supplementation on anthropometric and biochemical indices in patients with NAFLD, without considering any restriction on the language to detect the relevant citations. The PubMed search strategy was improved by consulting with an epidemiologist and was designed as a combination of the following search terms: “Vitamin D OR Cholecalciferol OR Calciol OR “Vitamin D 3” OR “Vitamin D3” OR Cholecalciferols OR Hydroxycholecalciferols OR “Hydroxyvitamins D″ OR Hydroxycholecalciferol OR Calcifediol OR “25−Hydroxyvitamin D 3” OR “25 Hydroxyvitamin D 3” OR “25−Hydroxycholecalciferol” OR Calcidiol OR Hydroxycholecalciferol OR Dedrogyl OR Hidroferol OR Calderol OR Dihydroxycholecalciferols OR “Dihydroxyvitamins D” OR “24,25−Dihydroxyvitamin D 3” OR Dihydroxyvitamin OR “24,25-Dihydroxyvitamin” OR “24,25-Dihydroxycholecalciferol” OR Dihydroxyvitamin OR Calcitriol OR “1 alpha,25-Dihydroxyvitamin” OR “1,25-Dihydroxyvitamin” OR “1,25-Dihydroxyvitamin” OR “1 alpha,25-Dihydroxycholecalciferol” OR “1,25−Dihydroxycholecalciferol” OR Dihydroxycholecalciferol OR Bocatriol OR Calcijex OR Decostriol OR MC1288 OR “MC−1288” OR “MC 1288” OR Osteotriol OR Renatriol OR Rocaltrol OR Silkis OR Sitriol OR Soltriol OR Tirocal” AND “Fatty Liver” OR “Fatty Liver” OR Steatohepatitis OR Steatohepatitides OR “Steatosis of Liver” OR “Visceral Steatosis” OR Steatoses OR Steatosis OR “Visceral Steatoses” OR “Liver Steatosis” OR “Liver Steatoses” OR “Non−alcoholic Fatty Liver Disease” OR “Non−alcoholic Fatty Liver Disease” OR “Nonalcoholic Fatty Liver Disease” OR NAFLD OR “Nonalcoholic Fatty Liver Disease” OR Nonalcoholic OR “Nonalcoholic” OR “Nonalcoholic Fatty” OR “Non−alcoholic Fatty” OR “Nonalcoholic Fatty Liver” OR “Nonalcoholic Fatty Livers” OR NASH OR “Nonalcoholic Steatohepatitis” OR “Nonalcoholic Steatohepatitides” OR Steatohepatitis OR “liver cirrhosis” OR cirrhosis OR cirrhosis. To improve the search strategy, the wild−card term “*” was implicated and the reference lists of relevant articles were hand−scanned. Any doubts were resolved through discussing with the corresponding author.

### Inclusion and Exclusion Criteria

Based on PICOS criteria, the aim of the present review study on patients with NAFLD (as population) was to evaluate the effect of vitamin D supplementation (as intervention) compared with placebo (as comparator) on anthropometric and biochemical outcomes (as outcome) in randomized interventional trials (as study design). Two independent reviewers initiated the assessment process of the collected papers and following inclusion criteria were considered (Younossi, 2019): randomized controlled trials (RCTs) with parallel design (Petta et al., 2016), studies that assessed the effects of vitamin D supplementation on patients with NAFLD. The exclusion criteria were (Younossi, 2019) being experimental study (Petta et al., 2016), lacking the essential data, for example, non−extractable or unconvertable data (Morvaridzadeh et al., 2021), studies without a suitable control group (Chalasani et al., 2012), trials with combined supplementation of nutrients or/and medicines besides vitamin D and also (Tolman et al., 2007) unpublished data, grey literature, conference abstracts, book chapters, and brief reports. The corresponding author was involved to solve disagreements.

### Data Extraction

Following data were extracted from the included trials by two independent investigators: first authors’ last name, publication date, country of study, study design, participants’ characteristics, dosage of the supplement, intervention duration, quality of trials, mean changes and standard deviations (SDs) for each outcomes in pre−treatment and post−treatment. The third reviewer solved disagreements.

### Risk of Bias Assessment

Two independent reviewers were assigned to qualify the included RCTs and explore the potential risk of bias for the following domains using the Cochrane Risk of Bias Tool: random sequence generation, allocation concealment, blinding of participants and personnel, blinding of outcome assessment, incomplete outcome data, and selective outcome reporting and other biases. “low”, “high” or “unclear” terms were used to grade each item (Higgins et al., 2011).

Each question was answered as low risk of bias (score = 1), high risk of bias (score = −1), or unclear (score = 0). Disagreements was resolved by the third author. Scores were summed and studies with scores −6−0, 1−3 and 4−6 considered as low, medium and high quality, respectively (Table 3).

### Statistical Analysis

A meta−analysis was performed using *STATA* software v13 (StataCorp, Texas) and a random−effects model was used to pool weighted mean difference (WMD) and corresponding 95% confidence intervals (CIs). The statistical heterogeneity among the studies was assessed using *I* ^2^ statistic (high ≥50%, low <50%) and Cochran’s Q−test (Higgins et al., 2019). In presence of high inter−study heterogeneity, a random−effects meta−regression was performed to find its potential sources. Subgroup analysis was done based on country (Iran vs. other), duration of treatment (>12 weeks, ≤ 12 weeks), and dosage (>25,000 IU/day, ≤ 25,000 IU/day). Mean changes of the interested outcomes and the relevant SDs were obtained executing following formulas, respectively: (mean at post intervention–mean at baseline), SD = √([SD2−baseline + SD^2^−post] − [2r × SD−baseline × SD−post]), assuming correlation coefficient (r) as 0.5 (Higgins et al., 2019). Sensitivity analysis was planned to assess the impact of each study on the pooled results by removing one study in a turn (Higgins et al., 2019). Potential publication bias was evaluated applying Egger regression and Begg’s tests. If there was a significant publication bias, “trim and fills” analysis was done to check the possible change of the significance of the result (Duval and Tweedie, 2000). A P−value < 0.05 was considered as statistically significant.

## Results

### Literature Search

A total of 3,916 potentially relevant articles were collected in the primary search (Figure 1). No additional article was detected via the hand searching. Among all of the references, 1840 were removed as duplicates. After title and abstract screening, 48 articles remained and after full-text review, 32 articles were excluded from the current study for different reasons. Thus, the final systematic review and meta-analysis was done on 16 papers (Table 1) (Sharifi et al., 2014; Foroughi et al., 2015; Barchetta et al., 2016; Foroughi et al., 2016; Nadjarzadeh et al., 2016; Sharifi et al., 2016; Lorvand Amiri et al., 2017; Sakpal et al., 2017; Dabbaghmanesh et al., 2018; Geier et al., 2018; Mansourian Hosseini et al., 2018; MOUODI et al., 2018; Hajiaghamohammadi et al., 2019; Hussain et al., 2019; Shidfar et al., 2019; Foroughi et al., 2014).

**TABLE 1:** Characteristics of the primary studies included in the meta−analysis.

Authors	Publication year	Country	Sample size case/control	Dosage of vitamin D	Type of intervention in control group	Follow−up duration	Significant out comes
Foroughi et al. (Foroughi et al., 2015)	2015	Iran	30/30	50,000 IU/week	Placebo	10 weeks	Vitamin D supplementation resulted in increased serum 25−hydroxy vitamin D concentration in the intervention group compared to the control group. Intake of vitamin D supplements led to a marginally significant decrease in fasting blood glucose in the intervention group compared to the control group. HOMA−IR decreased in the intervention group compared to the control group
Mansourian Hosseini et al. (Mansourian Hosseini et al., 2018)	2018	Iran	41/41	600,000 IU	Placebo	4 weeks	serum 25−hydroxyvitamin D significantly increased in the intervention group vs. the control. Total body fat decreased in the intervention group. while visceral fat was significantly different between the groups. Adiponectin, calcium, phosphors, and PTH levels increased, while liver enzymes, insulin, and HOMA−IR decreased in both. There were significant differences in mean changes of serum 25(OH) D, PTH, ALT, AST, ALP, and FBS between the groups after adjusting for baseline, TF and VF. Vitamin D injection did improve NAFLD severity
Hajiaghamoham madi et al. (Hajiaghamohammadi et al., 2019)	2019	Iran	40/40	50,000 IU/week	Placebo	10 weeks	A significant reduction in the variables including AST, ALT, total cholesterol, and LDL−C in both groups (*p* < 0.05). As for the two indices of FBG and TG, the control group exhibited significantly less variations (*p* < 0.05)
Shidfar et al. (Shidfar et al., 2019)	2019	Iran	Group1: (*n* = 37), group2 (*n* = 37) group3 (*n* = 36)	Group1: (*n* = 37) 1000 IU vitamin D + Group2: (*n* = 37) 1000 IU vitamin D + 500 mg/d as calcium Group3: (*n* = 36), placebo	Placebo	12 weeks	Reduction in serum ALT, AST, LDL−C/HDL−C, TC/HDL−C, and non−HDL C were significantly higher in the CaD compared with the P group
Geier et al. (Geier et al., 2018)	2018	Switzerland	30/30	2100 IU/daily	Placebo	18 weeks	Significant change was not observed in AST, AP, and GGT levels; significant change was observed in ALT
Dabbaghmanesh et al. (Dabbaghmanesh et al., 2018)	2018	Iran	Group1: 35, group2: 35, group3: 36	Group1:50,000 IU vitamin, Group2: 0.25 mg calcitriol, Group3: placebo	Placebo	12 weeks	serum alkaline phosphatase levels was significantly decreased from baseline levels in vitamin D3 group. Serum and gamma GGT level was also significantly decreased compared to the baseline levels in treatment group. There was no statistically significant difference between placebo and vitamin D3 group in terms of serum aminotransferase, ALP, serum GGT and lipid profile serum ALP levels was significantly decreased from baseline levels in calcitriol treated group. There was no statistically significant difference between placebo and calcitriol groups in terms of serum aminotransferase, ALP, serum GGT and lipid profile
Hussain et al. (Hussain et al., 2019)	2019	Iran	55/54	50,000 IU vitamin	Placebo	12 weeks	A reduction in HOMA−IR, liver enzymes ALT, AST, serum CRP and increase in serum adiponectin as compared to placebo group. However no significant changes were observed in both groups in terms of body weight, BMI, and serum lipid profiles
Taghvaei et al. (MOUODI et al., 2018)	2018	Iran	20/20	50,000 IU vitamin	Placebo	12 weeks	Mean BMI and serum liver enzymes decreased significantly in two groups. A significant improvement was observed in steatosis in both groups. No significant differences were observed between the two groups in steatosis when measured by CAP parameter
Foroughi et al. (Foroughi et al., 2016)	2020	Iran	30/30	50,000 IU vitamin	Placebo	10 weeks	Vitamin D supplementation resulted in an increase of serum 25(OH) D concentrations in inter group and intra−group in intervention group. At the end of the study, in the intervention group, TG and CRP reduced significantly compare with baseline. A significant increase was seen in calcium serum in the intervention group in comparison with baseline and compared with the placebo group
Nadjarzadeh et al. (Nadjarzadeh et al., 2016)	2010	Iran	36/37	Group1: low calorie diet+ 50,000 IU vitamin D, Group2: low calorie diet + placebo	Placebo	12 weeks	Significant reduction was observed in ALT and AP Significant change was not observed in AST levels
Amiri et al. (Lorvand Amiri et al., 2017)	2016	Iran	37/36	1000 IU/day	Placebo	12 weeks	Significant reduction in FPG, insulin, insulin resistance (by HOMA−IR) and TG concentrations and an increase in HDL.C was seen over the 12 weeks of study in each group. Adjusting to the baseline measurements, there was significant difference in FPG, HOMA−IR, serum insulin, TG and HDL.C among the groups after 12 weeks of the study. The calcium plus calcitriol group showed a significant decrease in ALT and FPG and increase in HDL.C level compared with the calcitriol group, adjusted to the baseline measures
Sharifiet al. (Sharifi et al., 2016)	2016	Iran	27/26	50,000 IU vitamin D	Placebo	every 14 days for 4 months	In both genders, serum 25(OH) D3 increased significantly. This increase was accompanied by significant decrease in serum TC and LDL−C in women. However, in men, vitamin D supplementation increased the levels of serum TC with no significant effects on LDL−C. Moreover, vitamin D significantly reduced serum hs−CRP in women. The median daily calcium intake in both genders was well below the dietary reference intake for adults
Sharifi et al. (Sharifi et al., 2014)	2014	Iran	27/26	50,000 IU/2 weeks	Placebo	12 weeks	In vitamin D supplementation compared to the controls, the median of serum 25(OH)D3 significantly increased. This increase accompanied by significant decrease in serum MDA and near significant changes in serum hs−CRP. Other variables showed no significant changes
Barchetta et al. (Barchetta et al., 2016)	2016	Italia	26/29	2000 IU/daily	Placebo	48 weeks	25(OH) vitamin D significantly increased in the treated group, no group differences were found in HFF, transaminases, CK18−M30, P3NP levels or FLI after 24 weeks. Vitamin D neither changed the metabolic profile nor the cardiovascular parameters
Foroughi et al. (Foroughi et al., 2016)	2014	Iran	30/30	50,000 IU vitamin D	Placebo	10 weeks	Vitamin D supplementation resulted in an increase of serum 25(OH) D concentrations in inter group and intra−group in intervention group. At the end of the study, in the intervention group, TG and CRP reduced significantly compare with baseline. A significant increase was seen in calcium serum in the intervention group in comparison with baseline and compared with the placebo group
Sakpal et al. (Sakpal et al., 2017)	2010	India	51/30	600,000 IU	Placebo	24 weeks	Significant improvement in serum ALT and serum adiponectin levels of intervention group

### Meta−Analyses

#### Effect of Vitamin D on Lipid Profile

**FIGURE 2:**
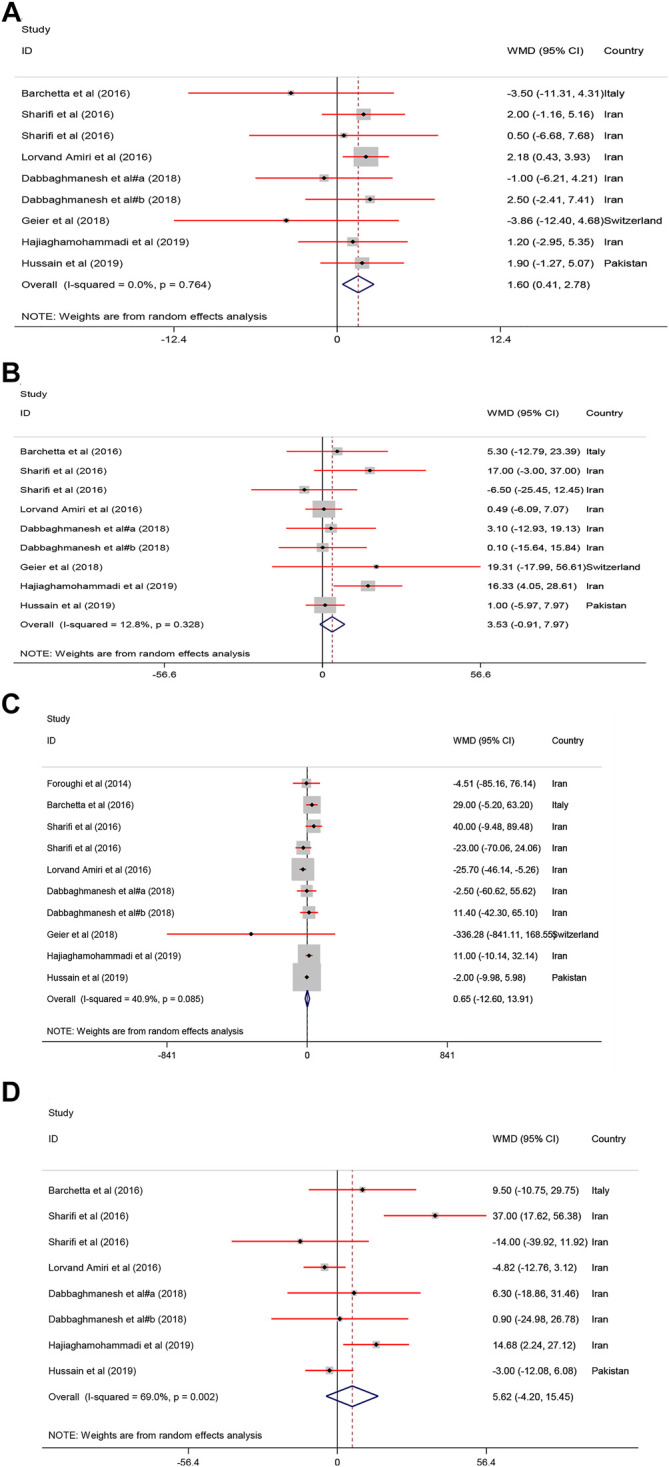
The lipid profile standardized mean differences estimates for **(A)** HDL−C, **(B)** LDL−C, **(C)** TG and **(D)** TC between intervention group (receiving vitamin D supplementation) and placebo groups.

A significant improvement of serum HDL−C [WMD = 1.60, 95% CI = (0.41, 2.78), *p* = 0.008, *I* ^2^ = 0.0%] was found following vitamin D intake compared to the controls. Serum levels of TG [WMD = 0.65, 95% CI = (−12.60, 13.91), *p* = 0.92, *I* ^2^ = 40.9%], TC [WMD = 5.62, 95% CI = (−4.20, 15.45), *p* = 0.26, *I* ^2^ = 69.0%] and LDL−C [WMD = 3.53, 95% CI = (−0.91, 7.97), *p* = 0.11, *I* ^2^ = 12.8%] were not significantly affected in the group receiving vitamin D (Figure 2).

#### Effect of Vitamin D on Anthropometric Indices

**FIGURE 3:**
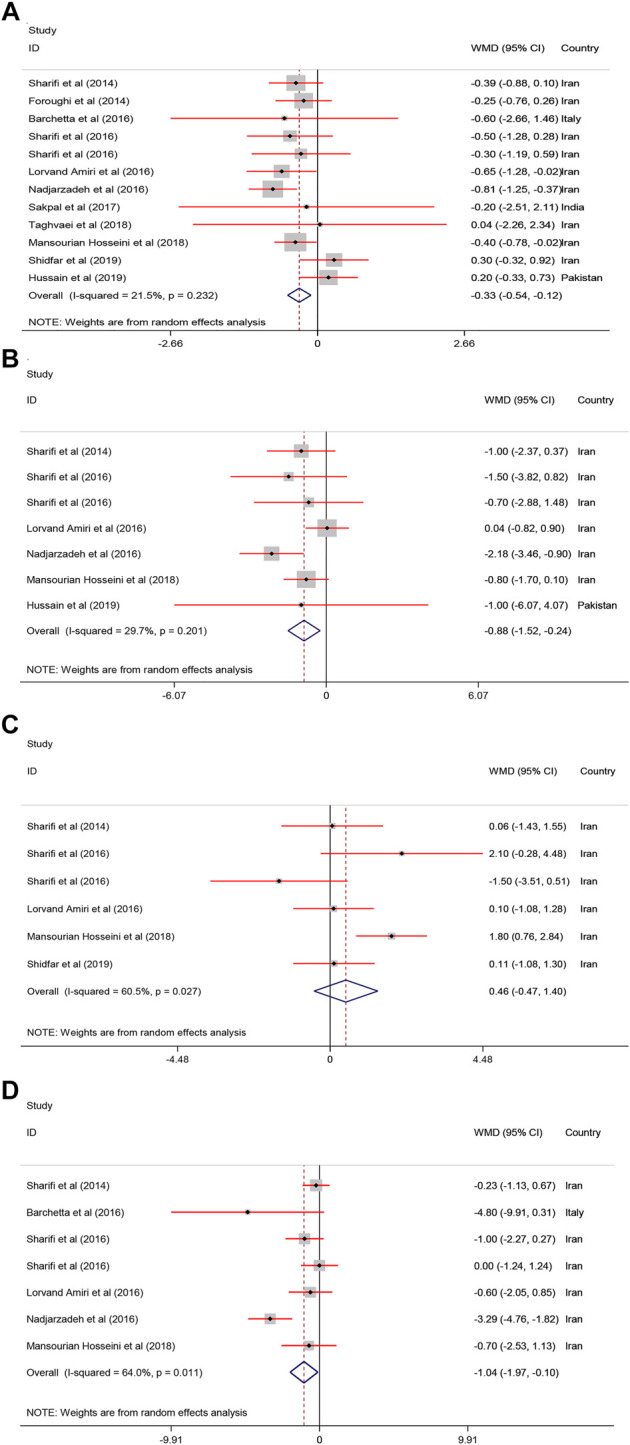
The anthropometric parameters standardized mean differences estimates for **(A)** BMI, **(B)** weight, and **(C)** body fat and **(D)** WC between intervention group (receiving vitamin D supplementation) and placebo groups.

Pooled results of the meta−analysis revealed a significant reduction in body weight [WMD = −0.88, 95% CI = (−1.52, −0.24), *p* = 0.007, *I* ^2^ = 29.7%], BMI [WMD = −0.33, 95% CI = (−0.54, −0.12), *p* = 0.002, *I* ^2^ = 21.5%], and WC [WMD = −1.04, 95% CI = (−1.97, −0.10), *p* = 0.02, *I* ^2^ = 64.0%] in the treatment groups compared with the controls. In contrast, body fat [WMD = 0.46, 95% CI = (−0.47, 1.40), *p* = 0.33, *I* ^2^ = 60.5%] was not changed following vitamin D supplementation (Figure 3).

#### Effect of Vitamin D on Liver Enzymes

**FIGURE 4:**
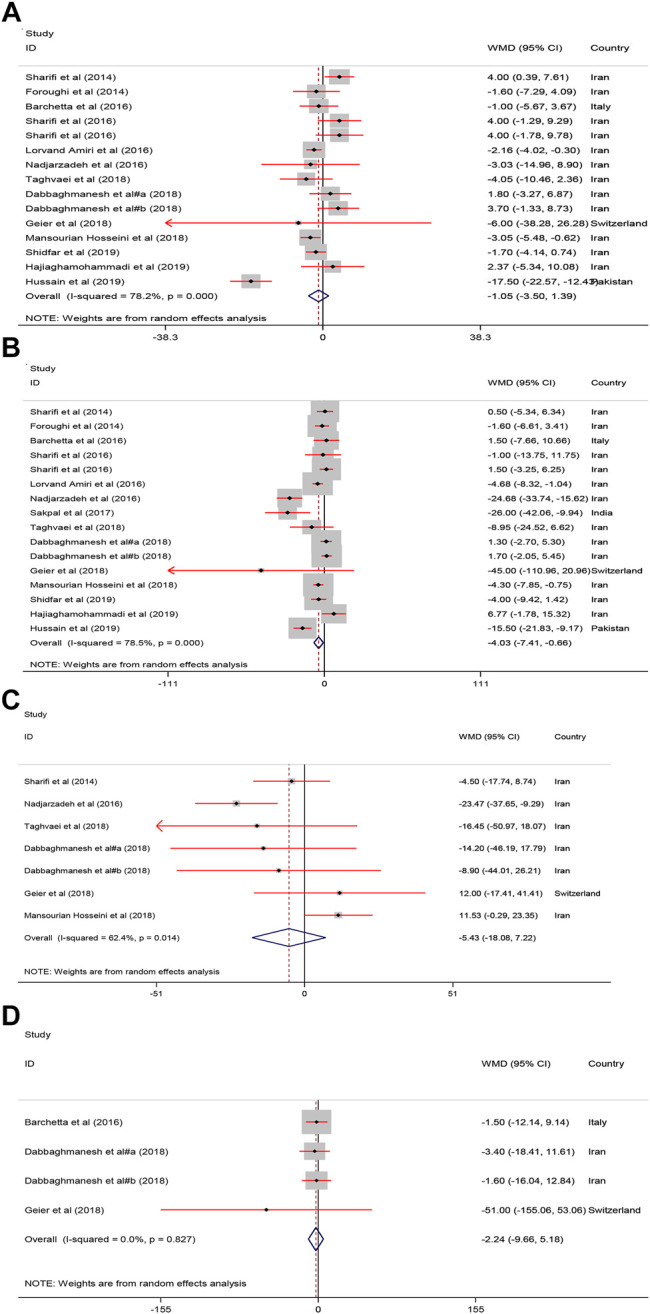
The Liver enzymes standardized mean differences estimates for **(A)** AST **(B)** ALT, **(C)** ALP and **(D)** GGT between intervention group (receiving vitamin D supplementation) and placebo groups.

Pooled estimate of 16 papers indicated a significant reduction of serum ALT [WMD = −4.03, 95% CI = (−7.41, −0.66), *p* = 0.01, *I* ^2^ = 78.5%] following vitamin D supplementation. However, serum AST [WMD = −1.05, 95% CI = (−3.50, 1.39), *p* = 0.39, *I* ^2^ = 78.2%], ALP [WMD = −5.43, 95% CI = (−18.08, 7.22), *p* = 0.40, *I* ^2^ = 62.4%], and GGT [WMD = −2.24, 95% CI = (−9.66, 5.18), *p* = 0.54, *I* ^2^ = 0.0%] were not affected by vitamin D supplementation (Figure 4).

#### Effect of Vitamin D on Glycemic Indices

**FIGURE 5:**
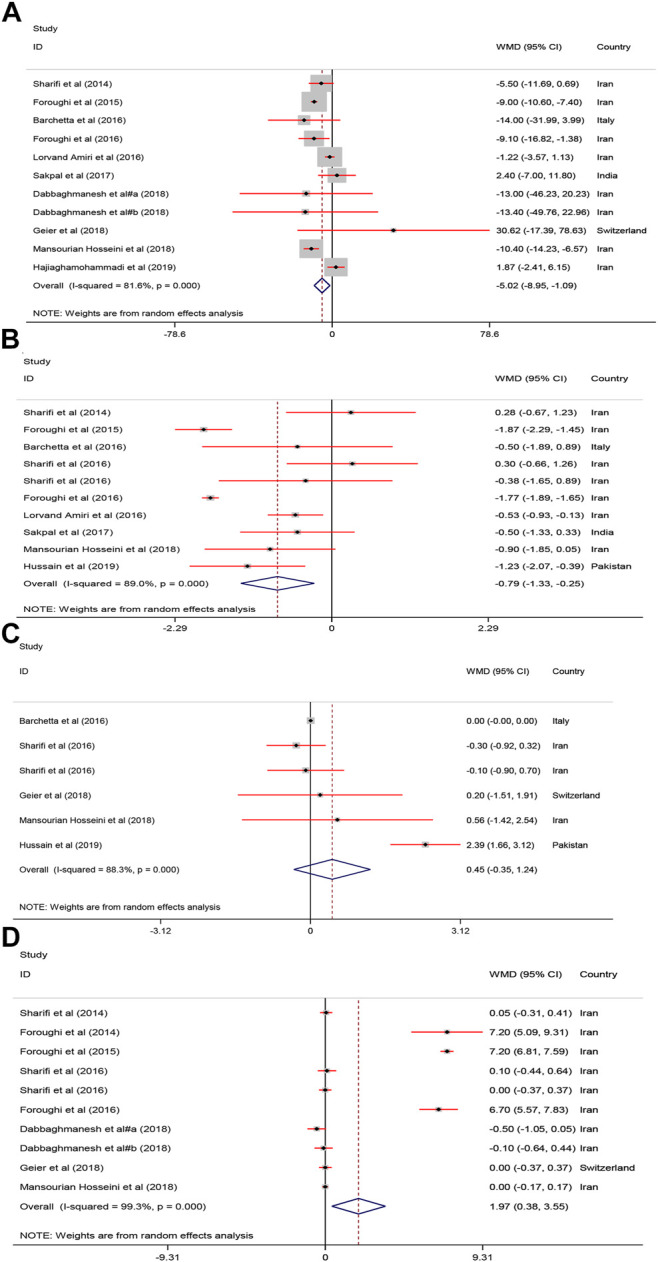
The FBS, HOMA, Adiponectin and calcium mean differences estimates for **(A)** FBG, **(B)** HOMA−IR, **(C)** Adiponectin and **(D)** calcium FBS: fasting plasma glucose; homeostasis model assessment of insulin resistance; RCTs, randomized controlled trials.

As shown in Figure 5, receiving vitamin D significantly improved serum FBS [WMD = −5.02, 95% CI = (−8.95, −1.09), *p* = 0.01, *I* ^2^ = 81.6%] and HOMA−IR [WMD = −0.79, 95% CI = (−1.33, −0.25), *p* = 0.004, *I* ^2^ = 89.0%] compared with to the controls.

#### Effect of Vitamin D on Adiponectin and Calcium

As shown in Figure 5, serum calcium was significantly increased after vitamin D supplementation [WMD = 1.97, 95% CI = (0.38, 3.55), *p* = 0.01, *I* ^2^ = 99.3%]. Adiponectin was not significantly affected [WMD = 0.45, 95% CI = (−0.35, 1.24), *p* = 0.26, *I* ^2^ = 88.3%] by vitamin D supplementation.

### Subgroup Analyses

As shown in Table 2 **,** serum HDL−C was significantly affected by vitamin D supplementation when subgroup analysis conducted by country (Iran), duration (≤12 weeks), and dose (≤25,000 IU). BMI was changed in country (Iran), duration (>12 weeks), and dose (≤25,000 IU) subgroups. Body weight was changed in studies conducted in Iran and also when used >25,000 IU doses of when which interventions last >12 weeks, serum FBS was significantly affected when studies performed in Iran, last ≤12 weeks, and used doses more than 25,000 IU vitamin D. HOMA−IR was significantly improved all subgroups, except in trials last more than 12 weeks.

**TABLE 2:** The results of subgroup analysis.

Subgroups		No. of study	WMD (95% CI)	*p*	Heterogeneity (*I* ^2^, *P*)
**TG**					
Country	Iran	7	−1.73 (−21.01, 17.55)	0.86	41.4%, 0.11
	Other	3	8.29 (−24.32, 40.90)	0.61	57.4%, 0.09
Duration	>12 weeks	4	14.03 (−25.09, 53.15)	0.48	49.7%, 0.11
	≤12 weeks	6	−3.87 (−15.21, 7.47)	0.50	26.0%, 0.23
Dosage	≤25,000 IU	5	1.31 (−34.31, 36.94)	0.94	69.2%, 0.01
	>25,000 IU	5	−0.23 (−7.53, 7.08)	0.95	0.0%, 0.83
**TC**					
Country	Iran	6	7.19 (−6.90, 21.27)	0.31	75.8%, 0.001
	Other	2	−0.16 (−10.43, 10.10)	0.97	17.9%, 0.27
Duration	>12 weeks	3	11.84 (−16.20, 39.89)	0.40	80.1%, 0.007
	≤12 weeks	5	1.39 (−6.58, 9.36)	0.73	46.1%, 0.11
Dosage	≤25,000 IU	4	7.02 −13.62, 27.66)	0.50	82.6%, 0.001
	>25,000 IU	4	4.45 (−5.80, 14.71)	0.39	0.16
**HDL−C**					
Country	Iran	6	1.82 (0.51, 3.13)	0.007	0.0%, 0.90
	Other	3	−0.22 (−4.17, 3.72)	0.91	27.3%, 0.25
Duration	>12 weeks	4	0.66 (−1.92, 3.25)	0.61	0.0%, 0.41
	≤12 weeks	5	1.84 (0.51, 3.18)	0.007	0.0%, 0.83
Dosage	≤25,000 IU	5	1.70 (0.25, 3.15)	0.02	0.0%, 0.43
	>25,000 IU	4	1.38 (−0.68, 3.44)	0.18	0.0%, 0.77
**BMI**					
Country	Iran	9	−0.40 (−0.61, −0.19)	<0.0001	15.1%, 0.30
	Other	3	0.13 (−0.37, 0.64)	0.60	0.0%, 0.73
Duration	>12 weeks	5	−0.40 (−0.77, −0.03)	0.03	0.0%, 0.99
	≤12 weeks	7	−0.29 (−0.61, 0.04)	0.08	56.2%, 0.03
Dosage	≤25,000 IU	7	−0.31 (−0.59, −0.02)	0.03	0.0%, 0.51
	>25,000 IU	5	−0.33 (−0.69, 0.03)	0.07	53.9%, 0.06
**Body weight**					
Country	Iran	6	−0.90 (−1.60, −0.20)	0.01	41.4%, 0.12
	Other	1	−1 (−6.07, 4.07)	−	−
Duration	>12 weeks	3	−1.03 (−2.07, 0.01)	0.05	0.0%, 0.88
	≤12 weeks	4	−0.89 (−1.94, 0.16)	0.09	62.6%, 0.04
Dosage	≤25,000 IU	4	−0.40 (−1.06, 0.27)	0.23	0.0%, 0.44
	>25,000 IU	3	−1.35 (−2.40, −0.31)	0.01	33.3%, 0.22
**ALT**					
Country	Iran	12	−2.37 (−5.46, 0.73)	0.13	74.0%, <0.0001
	Other	4	−13.61 (−27.33, 0.10)	0.05	77.6%, 0.004
Duration	>12 weeks	6	−2.41 (−8.67, 3.85)	0.45	59.6%, 0.03
	≤12 weeks	10	−4.69 (−8.79, −0.59)	0.02	83.6%, <0.0001
Dosage	≤25,000 IU	8	−2.72 (−6.67, 1.22)	0.17	56.3%, 0.02
	>25,000 IU	8	−4.98 (−10.41, 0.45)	0.07	87.0%, <0.0001
**AST**					
Country	Iran	12	0.10 (−1.70, 1.90)	0.91	54.9%, 0.01
	Other	3	−8.77 (−23.21, 5.67)	0.23	90.9%, <0.0001
Duration	>12 weeks	5	2.74 (0.44, 5.04)	0.02	0.0%, 0.46
	≤12 weeks	10	−2.59 (−5.45, 0.27)	0.07	79.9%, <0.0001
Dosage	≤25,000 IU	7	0.53 (−1.86, 2.91)	0.66	60.5%, 0.01
	>25,000 IU	8	−2.76 (−7.44, 1.91)	0.24	84.0%, <0.0001
**ALP**					
Country	Iran	6	−7.62 (−21.34, 6.09)	0.27	66.2%, 0.01
	Other	1	12.00 (−17.41, 41.41)	—	—
Duration	>12 weeks	2	−1.69 (−13.84, 10.46)	0.78	0.5%, 0.31
	≤12 weeks	5	−9.11 (−27.90, 9.67)	0.34	73.0%, 0.005
Dosage	≤25,000 IU	2	−1.69 (−13.84, 10.46)	0.78	0.5%, 0.31
	>25,000 IU	5	−9.11 (−27.90, 9.67)	0.34	73.0%, 0.005
**FBS**					
Country	Iran	8	−5.63 (−9.78, −1.48)	0.008	85.5%, <0.0001
	Other	3	−0.85 (−16.87, 15.17)	0.91	51.7%, 0.12
Duration	>12 weeks	4	−3.09 (−11.24, 5.07)	0.45	41.9%, 0.16
	≤12 weeks	7	−5.68 9–−10.34, −1.01)	0.01	87.5%, <0.0001
Dosage	≤25,000 IU	5	−2.20 (−6.33, 1.93)	0.29	31.9%, 0.20
	>25,000 IU	6	−6.87 (−11.81, −1.94)	0.006	78.8%, <0.0001
**HOMA−IR**					
Country	Iran	7	−0.79 (−1.44, −0.14)	0.01	91.7%, <0.0001
	Other	3	−0.81 (−1.35, −0.26)	0.004	0.0%, 0.43
Duration	>12 weeks	5	−0.12 (−0.58, 0.34)	0.60	0.0%, 0.61
	≤12 weeks	5	−1.30 (−1.88, −0.73)	<0.0001	89.4%, <0.0001
Dosage	≤25,000 IU	6	−0.35 (−0.65, −0.05)	0.02	0.0%, 0.49
	>25,000 IU	4	−1.68 (−1.96, −1.40)	<0.0001	39.0%, 0.17

**TABLE 3:** The results of risk of bias.

First author	Country	Random sequence generation (selection bias)	Alloation concealment (selection bias)	Blinding or participants and personnel (performance bias)	Blinding of outcome assessment (detection bias)	Incomplete outcome data addressed (attrition bias)	Selective reporting (reporting bias)	Other sources of bias (e.g. bias of study design, trial stopped early, extreme baseline imbalance and fraudulent)
Sharifi et al. (2014)	Iran	Unclear	Unclear	Low risk	Low risk	Low risk	Low risk	Low risk
Foroughi et al. (2014)	Iran	Unclear	Unclear	Low risk	Low risk	Low risk	High risk	Low risk
Foroughi et al. (2015)	Iran	Unclear	Unclear	Low risk	Low risk	Low risk	Low risk	Low risk
Barchetta et al. (2016)	Italy	Low risk	Low risk	Low risk	Low risk	High risk	Low risk	Low risk
Sharifi et al. (2016)	Iran	Unclear	Unclear	Low risk	Low risk	Low risk	Low risk	Low risk
Foroughi et al. (2016)	Iran	Low risk	Low risk	Low risk	Low risk	Low risk	Low risk	Low risk
Lorvandamiri et al. (2016)	Iran	Low risk	Low risk	Low risk	Low risk	Low risk	Low risk	Low risk
Nadjarzadeh et al. (2016)	Iran	Unclear	Unclear	Low risk	Low risk	Low risk	High risk	Low risk
Sakpal et al. (2017)	India	Unclear	Unclear	Unclear	Low risk	Unclear	Unclear	Low risk
Taghvaei et al. (2018)	Iran	Low risk	Low risk	Low risk	Low risk	Low risk	Low risk	Low risk
Dabbaghmanesh et al. (2018)	Iran	Low risk	Low risk	Low risk	Low risk	Low risk	Low risk	Low risk
Geier et al. (2018)	Switzerland	Unclear	Unclear	Low risk	Low risk	Low risk	Low risk	Low risk
Mansourian Hosseini et al. (2018)	Iran	Low risk	Low risk	Unclear	Low risk	High risk	Low risk	Low risk
Shidfar et al. (2019)	Iran	Low risk	Low risk	Low risk	Low risk	Unclear	High risk	Low risk
Hajiaghamohammadi et al. (2019)	Iran	Unclear	Unclear	Low risk	Low risk	Unclear	High risk	Low risk
Hussain et al. (2019)	Pakistan	Low risk	Low risk	Low risk	Low risk	Low risk	Unclear	Low risk

### Meta−Regression and Sensitivity Analysis

The results of meta−regression and sensitivity analysis indicated that total sample size was regarded as a source of inter−study heterogeneity for AST [Coefficient: −0.19, 95% CI = (−0.29, −0.08), *p* = 0.001]. Sensitivity analyses showed that pooled results of interested outcomes are not sensitive to removing any of included trials.

### Publication Bias

A significant publication bias was found for HOMA-IR, HDL-C and GGT. Hence, trim and fill analysis was done to detect the potential change of their results. Only the significance of HDL−C was changed and turned into non−significant.

## Discussion

To the best of the authors’ knowledge, this is the first study to systematically review and meta analyze the findings of intervention studies on the effect of vitamin D on a wide range of anthropometric and biochemical indices in patient with NAFLD. The results of the present study indicated that vitamin D supplementation significantly improved the body weight, BMI, WC, HDL-C, ALT, FBS, and HOMA-IR in the intervention group compared to the control group. However, no significant differences were found on body fat, TG, LDL-C, AST, and GGT. Subgroup analyses also showed that the effect of vitamin D on most indices was affected by study location, duration of study and dose used for vitamin D.

Some studies investigated the association between vitamin D supplementation with lipid profile in NAFLD. In line with this study, Dabaghmanesh et al. indicated that 12 weeks of vitamin D supplementation significantly increased HDL-C (Dabbaghmanesh et al., 2018). Another study found that supplementation with calcium combined with vitamin D improved serum ALT and HDL-C levels (Lorvand Amiri et al., 2017).

However, the results regarding the effect of vitamin D on lipid profile have been contradictory. Sharifi et al. found that vitamin D intake supplementation for 4 months significantly decreed LDL-C and total cholesterol in woman (Sharifi et al., 2016). A meta-analysis indicated that supplementation with vitamin D significantly increased LDL-C and had not significant effect on TG and HDL-C in patients with CVDs (Wang et al., 2012). One study found that vitamin D supplementation reduced LDL-C but no significant change was found on HDL-C (Hajiaghamohammadi et al., 2019). In another study supplementation with vitamin D3 as 50,000 IU per week for 12 weeks in NAFLD had no significant effect on lipid profile and body composition (Hussain et al., 2019). The effect of vitamin D on lipid profile in people with NAFLD may be different from its effect in healthy people or in people with other chronic diseases.

Some effects of vitamin D are exerted through its effect on increasing calcium absorption. Therefore, dietary calcium intake can also affect the role of vitamin D in determining serum lipid profile. Shidfar et al. found that vitamin D combined with calcium for 12 weeks reduced ALT, AST, LDL-C/HDL-C, TC/HDL-C, and increased non−HDL−C compared with group revived only vitamin D (Shidfar et al., 2019). Moreover, some studies suggested an inverse association between serum 25(OH)-D levels and TG (Chon et al., 2014). The underlying mechanisms of the effects of vitamin D on lipid profile has not been completely yet understood. The low levels of vitamin D can lead to hyperparathyroidism which can lower serum TG. On the other hand, lower vitamin D level can activate microsomal TG-transfer protein, which leads to increased TG. Vitamin D also regulates macrophage function in reverse cholesterol transportation and reduces inflammation and insulin resistance (Shidfar et al., 2019).

Regarding to anthropometric measurements, this meta−analysis found that vitamin D reduced body weight, BMI, and WC but no significant change was found in body fat. Hussain et al. investigated the association between vitamin D intake and BMI, body fat, and WC in NAFLD and reported that 50,000 IU vitamin D for 12 weeks had no significant effect on anthropometric indices (Hussain et al., 2019). Another study found that the injection of cholecalciferol (600,000 IU) reduced weight, WC, and BMI (Mansourian Hosseini et al., 2018). Sharifi et al. reported that the intervention group with 50,000 IU vitamin D3 for 4 month had lower weight, WC, and BMI compared with the controls (Sharifi et al., 2016). The reason for these differences may be the differences in doses of vitamin D supplementation and also differences in seum level of vitamin D. Doaei et al. reported that the serum level of vitamin D is inversely associated with obesity. However, short term changes of serum vitamin D was not related to changes in weight (Doaei et al., 2020).

The present study identified that ALT increased and some liver enzymes such as AST, ALP, GGT were not affected following vitamin D supplementation. Vitamin D receptor (VDR) is abundantly expressed on liver cells and this vitamin was reported to have an anti−inflammatory effect on liver (Bozic et al., 2016). One study found that 50,000 IU/wk vitamin D supplementation for 10 weeks reduced AST and ALT in the intervention group compared with the control group (Hajiaghamohammadi et al., 2019). The vitamin D supplementation for 48 weeks decreased liver enzyme in patients with NAFLD (Geier et al., 2018). The difference in the results of the effect of vitamin D on the serum level of liver enzymes may be due to the fact that the levels of liver enzymes is greatly affected by other lifestyle and dietary factors. Sakpal et al. indicated that vitamin D intake along with lifestyle modifications improved serum ALT levels (Jamali et al., 2013). Another placebo−controlled study found that 12−weeks hypocaloric diet combined with calcitriol improved ALT and AST in the intervention group compared with the control group (Amiri et al., 2016).

The levels of FBS and HOMA-IR were improved following vitamin D intake. In line with this study, one study indicated that calcitriol intake (25 mg) for 12 weeks improved HOMA−IR in patients with NAFLD (Amiri et al., 2016). A recent study reported that vitamin D intake increased insulin sensitivity (Inzucchi et al., 1998). Another study found a negative association between vitamin D supplementation and FBS (Pittas et al., 2007). In another study, calcitriol supplementation for a short term increased insulin sensitivity (Foroughi et al., 2016). One meta−analysis suggested that vitamin D administration could improve FBS and insulin resistance in patents with impaired glucose tolerance or HOMA−IR (George et al., 2012). However, the results regarding the effect of vitamin D on serum glucose indices were contradictory. One study reported that vitamin D had no association with FBS (Kitson and Roberts, 2012). Interestingly, Lind et al. found that long−term calcitriol supplementation for 2 years decreased HOMA−IR but had no effect on insulin (Lind et al., 1989). The short−term vitamin D intake was associated with lower FBS in renal disease patients while had no effect on fasting insulin levels (Sarathy et al., 2015). The underlying mechanism of the effect of vitamin D on blood glucose is not yet clear. Vitamin D deficiency increase serum parathyroid hormone levels (PTH) and the PTH plays an important role in NAFLD through increasing insulin resistance (IR) (Foss, 2009).

The results of the present study indicated that the level of adiponectin was not significantly affected by vitamin D supplementation. Some studies reported that patients with NAFLD had lower adiponectin concentration compared with the others and adiponectin can be considered as a predictor of the severity of hepatic steatosis (Targher et al., 2006; Nakano et al., 2011). In line with our study, two previous studies reported that vitamin D intake had no effect on serum adiponectin in people with type 2 diabetes and obese children (Patel et al., 2010; Belenchia et al., 2013). On the other hand, Nakano et al. reported that phototherapy and vitamin D increased adiponectin concentration in rats with NAFLD (Kitson and Roberts, 2012). Adiponectin is secreted from adipose tissue and could influence on metabolism of fat and glucose. Adiponectin increased liver insulin sensitivity and decreased glucose excretion from the liver which can lead to prevent of high blood sugar. It has been suggested that vitamin D regulates adiponectin gene expression in adipose tissue (Hajiaghamohammadi et al., 2019). It seems that many factors may influence the effects of vitamin D on serum adiponectin such as differences in doses of vitamin D supplementation, follow−up duration, and VDR gene polymorphisms. Moreover, the association between vitamin D intake and adiponectin may be influenced by other factors such as calcium intake and body weight. The results of a recent meta−analysis study indicated that vitamin D may promote secretion of adiponectin in subjects with diabetes and this effect may be potentiated if vitamin D intake is on daily basis and in combination with calcium but can be weakened by increasing BMI (Nikooyeh and Neyestani, 2021).

The present study found that vitamin D supplementation improved the level of serum calcium. In contrast with this result, Geier et al. found that adiponectin and calcium levels were not significantly different after vitamin D supplementation (Geier et al., 2018). Sharifi et al. showed that vitamin D supplementation has no effect on serum level of calcium (Sharifi et al., 2016). A recent study of more than 5,000 people found that taking a monthly vitamin D supplement did not alter serum calcium (Malihi et al., 2019). Serum calcium levels play an important role in the cardiovascular and muscular system and are maintained at a certain level by complex mechanisms (Fleet, 2017). Further studies on the effect of vitamin D on serum calcium levels and considering the interaction of various factors on serum calcium levels are needed.

## Conclusion

The present study showed that Vitamin D supplementation had significant effect on HDL-C, body weight, BMI, WC, serum ALT, serum FBS, HOMA-IR, calcium and no effect on TG, TC, LDL-C, AST, ALP, GGT, and adiponectin. Further studies are needed to identify the underlying mechanisms of the effects of vitamin D supplementation on the anthropometric and biochemical indices in patient with NAFLD.

## Data Availability

The raw data supporting the conclusions of this article will be made available by the authors, without undue reservation. Data may be made available upon request.

## Abbreviations

NAFLD, with non-alcoholic fatty liver disease; BMI, body mass index; WC, waist circumstance; ALT, alanine transaminase; FBS, fasting blood sugar; HOMA-IR, homeostatic model assessment for insulin resistance; TG: triglyceride.
